# Access to justice in State aid: how recent legal developments are opening ways to challenge Commission State aid decisions that may breach EU environmental law

**DOI:** 10.1007/s12027-021-00665-7

**Published:** 2021-05-10

**Authors:** Juliette Delarue, Sebastian D. Bechtel

**Affiliations:** ClientEarth, Brussels, Belgium

**Keywords:** State aid, Access to justice, EU environmental law, NGOs, Aarhus Convention

## Abstract

Access to justice in State aid matters is very limited. In particular, the admissibility test before the Court of Justice of the EU excludes private parties who are not market operators. The recent CJEU ruling in the *Hinkley Point C* case and the findings of the Aarhus Convention Compliance Committee call into question the adequacy of the current system. The findings demand an opening for non-market actors, including non-governmental organisations, to allege breaches of EU environmental law by the beneficiary of a State aid measure and consequently, the incompatibility of the aid measure with the internal market.

## Introduction

State aid measures are advantages under any form – such as loans, direct grants, tax rebates, financial support schemes – that a public authority may grant to certain enterprises (so-called undertakings) or economic sectors. Since it may affect trade or distort competition within the EU, State aid is in principle prohibited in the internal market and thus needs to be notified^1^ and authorised by the European Commission (‘Commission’) prior to being granted.^2^ It is rather frequent that Commission’s decisions on State aid^3^ are subject to petitions for annulment before the EU General Court (‘GC’), under Art. 263(4) TFEU;^4^ in few cases, they are also subject to references for preliminary rulings from national courts to the Court of Justice (‘Court’), under Art. 267 TFEU.^5^

The rarest are direct actions against Commission State aid decisions brought by non-market actors. There is no need to look for numbers: as further explained below, the admissibility thresholds for this category of applicants, which includes individuals and not-for-profit organisations (here together called ‘civil society’) is so high that it prevents them *de facto* from bringing any direct action to the Court of Justice of the European Union (‘CJEU’).

However, this does not imply that the granting or prohibition of State aid, as well as the compliance of aid with legal requirements, would only concern aid beneficiaries and competitors. Art. 106 and 107(2) and (3) TFEU provide that State aid may be granted when it: supports a service of general economic interest; has a social character and is granted to individual consumers; makes good the damage caused by natural disasters or exceptional occurrences (such as the COVID-19 pandemic); promotes the economic development of areas where the standard of living is abnormally low or where there is serious underemployment; remedies a serious disturbance in the economy of a Member State (again, like the COVID pandemic); facilitates the development of certain economic activities or of certain economic areas; or promotes culture and heritage conservation. These general policy objectives pursued by Member States when granting State aid undoubtedly have an impact on entire regions, on employment and social protection – not only on the enterprises receiving aid.

More importantly for the purposes of this paper, State aid decisions have a significant effect on environmental protection and compliance with the EU’s climate targets. State aid can either directly aim at improving environmental protection^6^ or can support economic activities that can have a harmful environmental impact, either because they are not complying with environmental law or have negative environmental externalities^7^ beyond those addressed by regulation. As explained below, this is not only a matter of contributing to environmental objectives but a matter of compliance with EU law. Nonetheless, the possibilities for non-market actors to challenge Commission decisions to improve or reject State aid are very limited.

On 17 March 2021, the Aarhus Convention Compliance Committee (ACCC)^8^ recommended the EU to amend its laws and practices to ensure that Commission State aid decisions that may breach EU law on the environment be subject to internal or judicial review, in accordance with Art. 9(3) and 9(4) Aarhus Convention.^9^ The findings demonstrate that the current possibilities to challenge EU State aid decisions are not only a barrier to the proper enforcement of EU law but also constitute a violation of international law.

In light of this background, this paper analyses the obligation of the Commission to comply with EU environmental law (Sect. 2) and the current possibilities for different applicants to enforce this obligation before the CJEU (Sect. 3). The paper then assesses the compliance of this situation with international law in light of the mentioned ACCC findings (Sect. 4) as well as the possibilities to remedy the existing non-compliance with international law (Sect. 5).

## State aid decisions may breach EU law on the environment

On 22 September 2020, the Grand Chamber of the Court ruled on appeal in the *Hinkley Point C* case.^10^ Austria had brought an action for annulment of Commission Decision (EU) 2015/658, by which the Commission approved three State aid measures notified by the United Kingdom in support of the Hinkley Point C nuclear power plant.

While Austria was unsuccessful on the merits of its claims, the judgment contains important clarifications of the general EU law principles governing the adoption of the Commission’s State aid decisions. Austria’s third ground of appeal challenged the GC’s judgment for having rejected its argument that “*the principle of protection of the environment, the precautionary principle, the ‘polluter pays’ principle and the principle of sustainability preclude the grant of State aid for the construction or operation of a nuclear power plant, on the ground that such an interpretation would be inconsistent with Art. 106a(3) of the Euratom Treaty*.”^11^

The Court of Justice concurred stating that, while these principles are not laid down in the Euratom Treaty, the Euratom Treaty does not preclude the application of rules of EU law on the environment to nuclear activities.^12^ The Court explicitly confirmed that “*the requirement to preserve and improve the environment, expressed in both the Charter and the FEU Treaty*”, such as Art. 37 of the Charter of Fundamental Rights, Art. 11 TFEU and Art. 194(1) TFEU, “*provisions of secondary EU law on the environment*”, such as the Environmental Impact Assessment Directive,^13^ as well as general principles of EU law are applicable.^14^

Based on the foregoing, the Court concluded that “*State aid for an economic activity falling within that sector* [nuclear power] *that is shown upon examination to contravene rules of EU law on the environment cannot be declared compatible with the internal market*”.^15^ Thus “*when the Commission checks whether State aid for an economic activity falling within that sector meets the first condition laid down in Art. 107(3)(c) TFEU (*…*) it must, as has been stated in paragraphs 44 and 45 hereof, check that that activity does not infringe rules of EU law on the environment. If it finds an infringement of those rules, it is obliged to declare the aid incompatible with the internal market without any other form of examination*”.^16^

The ruling explicitly refers to the nuclear power sector and the assessment of State aid under Art. 107(3)(c) TFEU, since they were at stake in the case. Nevertheless, these statements and their legal basis (Art. 11 TFEU, Art. 37 EU Charter of Fundamental Rights and Art. 194(1) TFEU) support a broader interpretation and a general obligation on the Commission to check that an activity complies with EU environmental law as a condition for it to receive State aid, regardless of the economic sector at stake. This is because this obligation stems from the general principle that “*decisions adopted by the Commission [on the basis of Art. 107(3)(c) TFEU] must ensure compliance with EU law*”.^17^

The judgment thereby clarifies, first, that the Commission must check, when authorising aid, whether the activity does not infringe rules of EU law on the environment; second, that the Commission cannot authorise State aid to an activity that breaches EU environmental law because such aid is necessarily incompatible with the internal market; and third, that this obligation is judiciable because the Court tested whether the Commission had complied with this obligation in the specific case.^18^ Conversely, a Commission negative decision refusing the grant of aid on the ground that an activity breaches environmental law when it is not the case should be subject to judicial review.^19^

Member State authorities and enterprises are subject to obligations relating to environmental and human health protection at large. National aid measures may contradict these requirements, whether they relate to the restoration of biodiversity, protection of habitats and species, preservation and improvement of water and air quality etc. Some examples of concerns that civil society has raised when intervening in CJEU cases concerning State aid are the support to electricity produced from indigenous coal in Spain, thereby crowding out other energy sources (*Castelnou Energia*); environmental impacts of increased road traffic of a rail and road infrastructure between Denmark and Germany (*the Fehmarn Link*, appeal pending) and the relief of the obligation for the Border Shops at the German-Scandinavian borders to charge a deposit for canned beverages and thereby pay the deposit VAT, as well as a relief of fines for violations of the deposit charging obligations, that risk reducing cans collection and recycling rates (*Dansk Erhverv*, pending). On the positive side, associations have also supported schemes for electricity produced from renewable energy sources (*Vent de Colère*). Besides these few court cases, individuals or NGOs also regularly file complaints and send observations to the Commission on State aid measures that may have an impact on the environment.^20^

Moreover, concerns as to the compliance of State aid with environmental law are likely to become more prevalent because the EU Green Deal seeks to ramp up the EU’s climate and environmental protection requirements. As far as climate protection is concerned, the EU and the Member States committed to reach certain levels of environmental and climate protection under international law such as the Paris Agreement and under EU law. In particular, the energy and climate targets for 2030 oblige the EU as a whole to reduce its greenhouse gas (GHG) emissions^21^ by at least 55% (while the European Parliament at the time of writing still insists on even 60%), increase the share of renewable energy to 32%, and increase energy efficiency by 32.5%.^22^ By 2050, the EU and the Member States committed to reach climate-neutrality, or the so-called “net-zero” GHG emissions.^23^ The national recovery and resilience plans, which are meant to help national economies recover from the consequences of the COVID-19 pandemic, should “*devote at least 37% of total expenditure to investments and reforms that contribute to the green transition, including biodiversity, or to addressing the challenges resulting therefrom*”.^24^

Having established the existence of this judiciable obligation, the remaining question is which applicants have the possibility to enforce the obligation in court. The following section addresses the existing EU legal framework as regards possibilities for administrative and judicial challenges directed against Commission’s State aid decisions.

## Existing legal avenues to challenge EU State aid decisions

In the EU legal order, the principle avenue to challenge acts and omissions of EU institutions are actions for annulment (Art. 263 TFEU) to the GC (Sect. 3.1 below). It is also possible to intervene in actions for annulment, in order to either support the applicant or the Commission, though this is not to be considered as a judicial avenue in its own right (Sect. 3.2 below). The action for annulment is rather complemented by the possibility for national courts to refer questions as to the validity of EU acts to the Court under Art. 267 TFEU, in cases where the validity of an EU act features in an existing national dispute (Sect. 3.3 below). In the State aid context, there is additionally the possibility to file a complaint to the Commission to inform it of an existing or potential breach (Sect. 3.4 below).

Leaving aside the special legal avenues available to Member States, this section explains to what extent these legal avenues are available to different applicants, contrasting in particular aid recipients and their competitors, on the one hand, with any other applicant, in particular civil society, on the other hand.

### Direct actions before the CJEU: annulment action (Art. 263 TFEU)

Direct actions for annulment of EU institutions decisions are provided for by Art. 263(4) TFEU, according to which “*Any natural or legal person may, under the conditions laid down in the first and second paragraphs, institute proceedings against an act addressed to that person or which is of direct and individual concern to them, and against a regulatory act which is of direct concern to them and does not entail implementing measures*.” Since Commission State aid decisions are only addressed to Member States, this admissibility test applies to all other applicants, including beneficiaries of the aid, their competitors and any third party.

As is well known, the CJEU has interpreted the terms “individual” and “direct” concern, very restrictively. According to long-established case law (the *Plaumann* case), “*persons other than those to whom a decision is addressed may claim to be individually concerned only if that decision affects them by reason of certain attributes which are peculiar to them or by reason of circumstances in which they are differentiated from all other persons and by virtue of those factors distinguishes them individually just as in the case of the person addressed*.”^25^ To the extent that a State aid scheme is considered a regulatory act, applicants still needs to demonstrate to be “directly concerned” by the decision, i.e. the contested scheme must directly affect their legal position.^26^

Under this test, the admissibility of aid beneficiaries is generally recognised for challenging individual aid measures and often for challenging aid schemes. Competitors of the aid beneficiary are considered “interested parties” and can therefore challenge Commission decisions on the ground of Art. 108(2) TFEU alleging a violation of their procedural rights, without any additional requirements.^27^ In order to challenge a Commission’s final decision adopted after a formal investigation based on Art. 106(2) or 107(3) TFEU, competitors needs to demonstrate instead that their competitive position is affected by the grant of aid.^28^

However, for all other applicants it is close to impossible to demonstrate being directly and individually concerned by the grant of aid because of the restrictive CJEU case law. Even the “lower” standard of “direct” concern, has in practice never been fulfilled by an applicant seeking to protect general interest such as the environment.^29^ This standard *de facto* excludes other categories of potential applicants including civil society from relying on Art. 263 TFEU to challenge EU State aid decisions.

### Interventions

Based on Art. 130 of the Rules of Procedures of the Court, a natural or legal person can apply to intervene in an existing action for annulment. Given that this possibility only exists if an action has already been filed by another applicant and because of the limited role an intervener can play (support claims filed by the applicant or the Commission etc), it is not equivalent to a right to challenge EU State aid decisions. However, it is mentioned here briefly to give a full picture of the avenues available to contest the legality of State aid decisions.

The admissibility threshold for such applications to intervene is high. First, having filed a complaint or observations to the Commission in the course of the State aid assessment procedure is not sufficient (for none of the categories of applicants).^30^ Moreover, the Commission in any event regularly considers that individuals and NGOs are not “interested parties” to file formal complaints.

Potential interveners must rather demonstrate that they have an interest in the solution of the case. For environmental NGOs, the admissibility standard is two-fold: “*for applications for leave to intervene submitted by environmental organisations, the requirement for a direct and existing interest in the result of the case means either that the scope of the activities of such organisations should coincide with that of the region and sector concerned by the proceedings before the Court or, where the scope of their activities is wider, that they should be actively involved in protection programmes or studies relating to the region and sector concerned, the viability of which could be jeopardised if the contested measure were to be adopted*”.^31^ The coincidence between the field of action and the region is assessed on a very strict geographical basis. For instance, whereas *Greenpeace España* was permitted to intervene against aid for electricity produced from indigenous coal plants in Spain, the pan-European scope of action of *ClientEarth* and *Stiching Greenpeace Council* (established respectively in the UK and the Netherlands) disqualified them from intervening in the same case.^32^ Even *Naturschutzbund Deutschland eV* (*NABU*), one of the largest environmental associations in Germany, did not fulfil this strict geographical coincidence criterion in the action against aid for the German-Denmark fixed link because it is also active outside of Germany.^33^ Conversely, Danish environmental NGO *Danmarks Naturfredningsforening* could intervene in a case relating to aid allegedly granted by Germany to shops selling beverages and situated near the German border with Scandinavian countries because “the purpose of the applicant to intervene *is confined* to protection of the environment *in Denmark*”.^34^

Alternatively, NGOs must demonstrate that “*they are actively involved in protection programs or studies concerning the region and the sector concerned, the viability of which could be jeopardised if the contested measure were to be adopted*”. This criterion as well is narrow: it is not sufficient for NGOs to demonstrate that they conduct numerous activities against electricity production from coal in various EU countries if the contested aid relates only to coal in Spain, for example.^35^ However engaging in campaigns, commissioning studies and reports specifically related to the contested project and exposing its impacts, support the admissibility of an NGO intervention.^36^

In recent orders though, the GC did not offer this alternative criteria and merely required that “*first, the remit of those organisations, as derived from their objective laid down, as the case may be, in their articles of association, has a direct link with the subject matter of that case and, second, that that case raises questions of principle which are liable to affect the interests defended by the organisations in question*”.^37^ Pursuant to this order, *Aktionsbündnis gegen eine feste Fehmarnbeltquerung*, established in Germany, was left to intervene in the action against the Commission decision authorising aid for the construction of the Fehmarn Link between Denmark and Germany, because “*the purpose of that association*, as stated in its statutes, is to oppose the implementation of the project at issue”.^38^

The General Court estimates that these criteria offer “*a broad interpretation of the right of associations to intervene, intended to facilitate assessment of the context of such cases whilst avoiding multiple individual interventions which would compromise the effectiveness and proper course of the procedure*”.^39^ However, as the analysis above shows, the current legal situation is far from clear.

### Preliminary rulings

The admissibility of an applicant to refer a question for preliminary ruling to the Court in a State aid matter depends, primarily, of their admissibility to bring an action before a national court. A good example of a preliminary ruling reference brought by an association and individuals before a French court is the *Vent de Colère* case, relating to support schemes for renewable energy production in France.^40^ However, according to the principle of procedural autonomy, admissibility before national courts depends on national procedural rules and is not harmonised in the EU.

In practice, for applicants other than aid recipients or competitors, standing at national level to challenge national aid measures, in order to then obtain a preliminary reference to the Court to contest the validity of the Commission State aid decision is far from guaranteed. A recent study for the Commission concludes that “*broad legal standing is granted by law and in practice in less than half of the Member States* (*13 out of then 28*).”^41^ The same study confirms that judges are often hesitant to refer questions as to the validity of EU acts,^42^ national court proceedings involving preliminary questions take many years^43^ and are often prohibitively expensive,^44^ all of which constitute formidable barriers to the effective use of this mechanism. Preliminary questions on State aid matters are particularly rare.^45^

### Complaints to the Commission

In accordance with Art. 24(2) Council Regulation 2015/1589, any interested party may submit a complaint “to inform” the Commission of alleged unlawful aid or misuse of aid.^46^ The Commission then follows up on the complaint and retains full control over the procedure. The complaint is therefore not an administrative or judicial remedy but a formalised possibility to inform the Commission of a possible breach, with a view to the Commission relying on Art. 108 TFEU to put a halt to the breach, if it considers that appropriate. It is not a possibility to challenge the Commission’s decisions in themselves, with a subsequent possibility to appeal to the courts.

Having clarified that, access to the complaint procedure is also limited to “interested parties”, as opposed to for instance a regular complaint to the Commission with the aim of starting infringement proceedings under Art. 258 TFEU. Interested parties are defined in Art. 1(h) Regulation 2015/1589 as “*any Member State and any person, undertaking or association of undertakings whose interests might be affected by the granting of aid, in particular the beneficiary of the aid, competing undertakings and trade associations*”. While this definition is in principle phrased in a broad and open manner (“any person … whose interests might be affected”), the Commission regularly denies requests from environmental NGOs on this basis stating that they are not “interested parties” for the purposes of the Regulation, for their market position not being affected by the grant of aid. The Commission nevertheless suggested, in the course of the communication before the Aarhus Convention Compliance Committee mentioned below, that environmental NGOs could be considered interested parties, and their complaints admissible, if they allege breaches of environmental law by the beneficiary of aid.^47^

## The resulting failure of the EU to comply with the Aarhus Convention

As the previous section demonstrates, the existing possibilities to challenge the Commission’s State aid decisions, as interpreted by the Commission itself (in the case of complaints) and the CJEU (as regards actions for annulment), are in practice mostly available to either Member States or aid recipients and their competitors. Other applicants will usually fail for various reasons to challenge the Commission’s State aid decisions before the CJEU.

The practical effect of this situation is that the above-mentioned obligation of the Commission to ensure that aid measures comply with environmental law is usually not enforced in court. Breaches of environmental law by the aid beneficiary are rarely, if ever, raised by aid beneficiaries and their competitors. These categories of applicants generally raise classic arguments relating to the assessment of the necessity, adequacy and proportionality of aid, its incentive effect and its effect on trade and competition in the internal market. Aid recipients are interested to safeguard financial support for their investments and activities, whereas competitors are eager to avert distortions of competition caused by the aid. Potential breaches of environmental law by aid beneficiaries are therefore not usually part of the judicial debates and thus risk to remain unsanctioned.

In fact, based on information submitted by the Commission in the proceedings before the ACCC further discussed below, there has so far been no action for annulment brought by civil society to challenge a State aid decision for failing to ensure compliance with EU environmental law. The authors are equally aware of only one preliminary reference case brought by individuals and associations, in the *Vent de Colère* case.

Generally, lack of enforcement is perhaps the greatest challenge of EU environmental law today.^48^ The Commission has estimated the cost of poor implementation of EU environmental law at around €50 billion a year.^49^ This by now a rather old figure and is likely to constantly increase as global environmental crises worsen. This persistent issue has been a core motivating factor for States to agree already in the 1992 Rio Declaration that: “Environmental issues are best handled with participation of all concerned citizens” and further that “Effective access to judicial and administrative proceedings, including redress and remedy, shall be provided.”

In Europe, this principle finds its legal expression in a binding treaty, the Convention on Access to Information, Public Participation in Decision-Making and Access to Justice in Environmental Matters done at Aarhus, Denmark, on 25 June 1998 (the Aarhus Convention). All EU Member States as well as the EU itself are Parties to this Convention. As its title indicates, the Convention provides for specific obligations in relation to three pillars, namely access to information, public participation and access to justice. As part of the access to justice pillar, Art. 9(3) gives “members of the public”, which include individuals and environmental NGOs (Art. 2(4)), the right to challenge acts and omissions from public authorities that contravene national law related to the environment.

As regards the acts of EU institution, the Aarhus Convention is implemented in EU law by Regulation (EC) No 1367/2006 (the Aarhus Regulation).^50^ Strikingly, State aid decisions adopted by the Commission are explicitly excluded from the scope of the Aarhus Regulation. Specifically, Art. 2(2)(a) Aarhus Regulation provides that “*Administrative acts and administrative omissions* [that are normally subject to internal or judicial review] *shall not include measures taken or omissions by a Community institution or body in its capacity as an administrative review body, such as under: (a) Articles 81, 82, 86 and 87 of the Treaty (competition rules)*”.^51^

The direct consequence of this exclusion is that members of the public cannot rely on their Aarhus Convention rights before the CJEU to challenge Commission decisions on State aid that may affect the environment. At least this is the interpretation of the GC: in the order in *Castelnou Energia*, the GC first stated that the Aarhus Regulation excludes judicial review of State aid decisions based on its Art. 2(2)(a). Second, “*a compliant interpretation* [of the EU admissibility criteria with Art. 9(3) Aarhus Convention] *cannot ignore the existence of the conditions explicitly laid down by the treaty and its implementing provisions for actions brought by this type of organization. To adopt the interpretation of the Aarhus Convention of the applicants to intervene, according to which in substance any organization for the defense of the environment could, by its sole purpose, intervene in a dispute, would precisely amount to ignoring the condition of justification of ’an interest in the solution of the dispute provided for by Art. 40 of the Statute of the Court to distinguish the conditions of intervention of States and institutions from those of other persons*” (point 26).

However, as mentioned above, the ACCC recommended in its findings of 17 March 2021 that the EU amend its laws and practices to ensure that State aid decisions that may breach EU law on the environment be subject to internal or judicial review, in accordance with Art. 9(3) and 9(4) Aarhus Convention.

The communication that gave rise to these findings had been filed by two Austrian NGOs (Global 2000 and Oköburö) in reaction to the impossibility for NGOs to meet the CJEU’s admissibility standard to challenge the Commission’s decision authorising aid measures for the construction of the Hinkley Point C nuclear power plant.^52^ In substance, the NGOs were arguing that the construction of a new nuclear power plant would breach EU law on the environment including the principles of protection of the environment, the precautionary principle, the polluter pays principle and the principle of sustainability; similar to the arguments made by Austria in its action before the CJEU in Case C-594/18P. Since the Commission’s decision was allowing the UK to grant aid to a project in breach of these rules and principles, they argued, this decision should be susceptible of internal or judicial review under Art. 9(3) Aarhus Convention.

The Commission, representing the EU, raised various arguments in order to defeat the claims of the communicants. Most importantly, the Commission argued that (a) State aid decisions are adopted by the Commission in its capacity as a review body and not as an administrative authority and that they would therefore fall outside the scope of the Convention; (b) these decisions are not susceptible to breach environmental law; (c) the declaration filed by the EU upon accession to the Convention would clarify that the obligation to provide for access to justice did not fall on the EU institutions but on the Member States and (d) the EU has a complete system of judicial remedies with Art. 263(4) and Art. 267 TFEU that allow for an adequate review of State aid decisions by the CJEU.

The ACCC disagreed on all four points. As regards point (a) the Committee had already established in earlier findings that the Aarhus Convention does not provide for an exception for acts taken in the capacity of an administrative review body, only for acts taken in a judicial or legislative capacity.^53^ The Committee further clarified that the Commission was acting in State aid matters more akin to a permitting body, rather than conducting an administrative review.^54^ Concerning (b) the possibility for State aid measures to comply with EU environmental law, the Committee referred to the Court judgment in *Hinkley Point C* to clarify that there was a clear obligation on the Commission to ensure compliance with EU environmental law.^55^ Art. 9(3) Aarhus Convention therefore applies to Commission State aid decisions. The ACCC further clarified that the EU’s declaration upon access to the Convention (c) only exempted the EU from ensuring access to justice for acts adopted by Member State authorities, not for those adopted by EU institutions.^56^ Finally (d), the ACCC considered that all the above-mentioned legal avenues were inadequate to challenge EU State aid decisions, a confirmation of its earlier findings on communication ACCC/C/2008/32 (Part I), rendered already in 2011.^57^

In a nutshell, the ACCC found that the remedies available to members of the public (individual and NGOs) are not sufficient to ensure the full application of Art. 9(3) Aarhus Convention.

## The way forward: consequences and remedies to these inconsistencies

In light of the EU’s failure to comply with the Aarhus Convention, as interpreted by the ACCC, the EU will need to amend its laws and practices, including the admissibility standard before the CJEU, to allow members of the public to challenge Commission State aid decision that may breach EU environmental law.

However, although it is a primary requirement, it is not sufficient to simply amend Art. 2(2)(a) Aarhus Regulation to remove the exclusion of State aid decisions from the scope of administrative acts that are subject to internal or judicial review. To be truly effective, access to justice for members of the public requires further changes to procedure and practice.

### Internal review of State aid decisions

As far as judicial review of State aid decisions is concerned, it is now clear that the CJEU should adopt a more permissible admissibility standard for civil society applicants, both for direct actions and interventions.

For internal review, the ACCC findings of 17 March 2021 call on the EU to provide “*internal or judicial review*” of State aid decisions.

Based on Art. 10 Aarhus Regulation, “*Any non-governmental organisation which meets the criteria set out in Art. 11 is entitled to make a request for internal review to the Community institution or body that has adopted an administrative act under environmental law or, in case of an alleged administrative omission, should have adopted such an act*”. Currently, a NGO must make such a request within six weeks after adoption of the decision. The Commission proposal to amend the Regulation would expand this to eight weeks. The applicant NGO is then able to institute proceedings before the CJEU against the internal review decision within two months based on Art. 263(4) TFEU, since is the addressee of the internal review decision (Art. 12 Aarhus Regulation).

Such review would have several practical implications on the State aid procedure. Pursuant to a request for internal review, the Commission would need to re-examine the State aid measure in light of allegations and evidence of breaches of environmental law by the activity receiving the aid. If the Commission concludes that the activity does not breach environmental law and that its original decision must stand, the applicant could challenge this act before the GC. If, on the contrary upon review, the Commission finds a breach of environmental law, it should in principle withdraw its original decision and adopt a new one.

In the authors’ opinion, in practice this new decision should be one to open a formal investigation, because there would be doubts as to the compatibility of the measure with the internal market.^58^ This is because, if the Commission finds during the internal review procedure that environmental law has been breached, or suspects it could have been, the Commission can neither authorise the measure because of the principles in the *Hinkley Point C* ruling (points 44-45 and 100), nor can it immediately adopt a negative decision, based on the Procedural Regulation. The opening of a formal investigation allows interested third parties to submit comments on the compatibility of the aid measure with the internal market, but also allows the Commissions to complete its investigation by collecting additional evidence and request information directly from the potential aid beneficiaries or other market operators.^59^ This is thus the opportunity to confirm whether the supported activity breaches EU environmental law.

### Increase transparency: a win-win-win

In order to minimize the number of internal review requests that the Commission may receive in the future if such an amendment was implemented, the Commission should increase transparency of this decision-making process. An in-depth and independent assessment of compliance of aid beneficiaries with environmental law, with clear statements of reasons and details on the evidence analysed would certainly reduce the risk of internal review applications based on allegations that the Commissions had actually already verified. This would also reduce the risk of direct actions against the State aid decisions based on Art. 108(2) TFEU. A win-win-win for the Commissions, third parties, and compliance.

It should be clarified that providing details on an activity’s compliance with its environmental law obligations would not necessarily breach confidentiality of business secrets or of the exchanges between the Commissions and the Member States. Stating reasons for a decision is a legal requirement subject to a strict judicial control (Art. 296 TFEU) and is a good practice in a decision-making process. The Commission is generally not hesitant to detail economic information that ground its reasoning on the necessity and proportionality of an aid measure, for example; genuine business secrets such as financial figures are usually redacted. When the Commission finds that an aid scheme (that would benefit multiple enterprises) would not ensure that only those activities that comply with their environmental law obligations can receive aid; or when a particular legislation is not adequately implemented in national law^60^ thus not ensuring compliance by aid beneficiaries, the authors argue that such information should be deemed to be of an overriding public interest, as per the case law criterion, and made public in the State aid decisions. For individual aid measures, the State aid decision should contain at least sufficient information to identify what types of documentation (environmental impact assessments, environmental permits, business plans, environmental impact reporting etc.) have been analysed by the Commission.

### Interventions

Additionally, the standard for letting NGOs intervene in State aid cases should be broadened. Even if non-market actors are able to request an internal review of EU State aid decisions, they should also be able to intervene in challenges brought by market actors to defend the proper application of EU law. As mentioned at the outset, EU State aid decisions may also fail to comply with EU environmental law for failing to recognize their positive contribution to the achievement of the objectives of environmental legislation. In such a case, an applicant may wish to intervene in support of a potential aid recipient. In other cases, an intervener may seek to defend the original decision adopted by the Commission for defending the correct application of environmental law. This should be taken into consideration in the interpretation of the admissibility standard.

## Conclusion

The ACCC findings establish that the judicial remedies offered by the EU for challenging Commission State aid decision breach international law. This is a big and welcome step: it recognises the potential for State aid decisions not to ensure compliance of activities with their environmental law obligations, and the need to offer adequate review remedies.

Currently, the EU Aarhus Regulation is under revision. This is the opportunity to align the Regulation with the Convention by removing the exclusion of State aid decisions from Art. 2(2)(a) and ensuring that acts of general scope can be challenged. A tremendous increase of direct actions against Commission State aid decisions is not to be expected though: the number of actions brought by enterprises is rather low compared to the number of decisions the Commission adopts each year and other applicants are likely to appeal to the Commission (and to the GC) even more rarely.

Perhaps the main advantage of such an amendment would rather be the added recognition of the obligation on the Commission to verify, and state reasons for its assessment, compliance of activities with environmental law prior to authorising an aid measure. There is certainly room for the Commission to improve and systematise this assessment. If adequately performed, this assessment has the potential to bring a great benefit for the health of Europeans and the environment they live in.

## Footnotes

1 Art. 107 and 108 TFEU. Aid measures falling within the General Block Exemption Regulation are exempted from notification and can be paid directly subject to meeting the regulatory conditions.

2 Providing the readers with a general background on the notion and regime of State aid would need extensive developments that fall outside the scope of this paper. The Commission Notice on the Notion of State aid (C/2016/2946, OJ C 262 of 19.7.2016, p. 1–50) can offer useful guidance to understand the concepts and type of public support that qualify as State aid. Procedural rules can be found in Council Regulation (EU) 2015/1589 of 13 July 2015 laying down detailed rules for the application of Art. 108 of the Treaty on the Functioning of the European Union, OJ L 248 of 24.9.2015, p. 9–29.

3 The Commission may take several types of decisions: decisions not to raise objections; decisions to open formal investigations, positive decisions, negative decisions with or without recovery of aid. Unless specified, there are together referred as ‘Commission decisions’ or ‘State aid decisions’ in this paper.

4 In 2019, out of 848 new direct actions, 157 concerned State aid or competition law; 278 State aid cases were pending and 102 State aid or competition law cases were completed. See CJEU Annual Report 2019, at: https://curia.europa.eu/jcms/upload/docs/application/pdf/2020-05/ra_pan_2019_interieur_en_final.pdf.

5 Commission Study on the enforcement rules and decisions of State aid by national courts (2019), available at: https://ec.europa.eu/competition/publications/reports/kd0219428enn.pdf. Figure 12 reveals that only one “indirect challenge of a Commission decision via CJEU preliminary rulings” in Belgium and four in France were identified since 2014 by the authors of the study.

6 The main relevant guidelines are the State aid guidelines for environmental protection and energy, the Agriculture and Forest Guidelines. This is without prejudice of the authors’ opinion on the fitness of these guidelines to achieve higher levels of environmental protection.

7 Such as GHG emissions, pollution of air, water and soil, loss of biodiversity.

8 The Aarhus Convention Compliance Committee is the entity who reviews compliance of the Parties with the provisions of the Convention in a non-confrontational, non-judicial and consultative manner. It issues non-binding recommendations that then need to be adopted by the Parties in a Meeting. See Aarhus Convention, Art. 15.

9 See the ACCC’s findings in Communication ACCC/C/2015/128 and all submissions from the Communicant, Observers (including ClientEarth) and the European Commission at: https://unece.org/env/pp/cc/accc.c.2015.128_european-union.

10 Judgment of 22.09.2020, *Austria v. Commission*, C-594/18P, ECLI:EU:C:2020:742.

11 Ibid, para. 34.

12 Ibid, para. 41.

13 Directive 2011/92/EU of the European Parliament and of the Council of 13 December 2011 on the assessment of the effects of certain public and private projects on the environment (OJ 2012 L 26, p. 1).

14 Judgment of 22.09.2020, *Austria v. Commission*, paras 42-44.

15 Ibid, para. 45.

16 Ibid, para. 100.

17 Ibid, para. 20.

18 See ibid, paras 48-50.

19 The authors are not aware of any such example.

20 ClientEarth, for example, regularly files complaints against State aid that support fossil fuels extraction, production or use. Complaints from civil society are regularly deemed inadmissible by the Commission for not demonstrating that the complainants’ market position would be affected by the grant of aid: such complaints are then treated as market information.

21 From 1990 levels.

22 The legally binding targets for Member States by 2020 were a reduction of GHG emissions of 20%, a share of renewables of 20% and an increase in energy efficiency of 20%.

23 Commission Proposal for a Regulation establishing the framework for achieving climate neutrality and amending Regulation (EU) 2018/1999 (European Climate Law), COM/2020/80 final; European Council conclusions of 12.12.2019, CO EUR 31 CONCL 9.

24 National recovery and resilience plans shall be prepared by Member States, and approved by the Commission, for the implementation of the new EU Recovery and Resilience Facility Regulation. See Regulation (EU) 2021/241 of the European Parliament and of the Council of 12 February 2021 establishing the Recovery and Resilience Facility, OJ L 57, 18.2.2021, p. 17–75.

25 Judgment of 15.07.1963, *Plaumann & Co. v Commission of the European Economic Community*, Case 25-62, ECLI:EU:C:1963:17.

26 Compare Joined cases C-622/16 P to C-624/16 P, *Scuola Elementare Maria Montessori Srl & Pietro Ferracci v Commission*, ECLI:EU:C:2018:873, paras 42-44.

27 Judgment of 3.09.2020, *Vereniging Gelijkberechtiging Grondbezitters e*.*a. v. Commission*, C-817/18 P, ECLI:EU:C:2020:637, point 51; see previously: Judgment of 18.11.2010, *NDSHT v. Commission*, C-322/09 P, EU:C:2010:701, point 59.

28 See e.g. Judgment of 3.12.2014, *Castelnou Energia S*.*L. v. Commission*, T-57/11, ECLI:EU:T:2014:1021, point 28.

29 Compare, T-600/15, *PAN Europe, Bee Life and Unapii v Commission*, ECLI:EU:T:2016:601, in which an environmental NGO sought to challenge a plant authorization because of its effects on bees. The organizations were not considered directly concerned by the decision.

30 Order of the General Court of 6.11.2012 in case T-57/11, *Castelnou Energia v. Commission*, ECLI:EU:T:2012:580; Order of 30.11.2016, T-630/15, *Scandlines Danmark ApS and Scandlines Deutschland GmbH v. Commission*, point 31.

31 Order of 21.10.2014, *Bayer CropScience v Commission*, T-429/13, EU:T:2014:920, paragraph 73 and the case-law cited; Order of the General Court of 6.11.2012 in *Castelnou Energia v. Commission*, point 10; Order of 30.11.2016, *Scandlines Danmark ApS and Scandlines Deutschland GmbH v. Commission*, point 17; Order of 10.09.2019, T-47/19, *Dansk Erhverv v. Commission*, ECLI:EU:T:2019:663, point 13.

32 Order of the General Court of 6.11.2012, *Castelnou Energia v. Commission*, point 21.

33 Order of 30.11.2016, *Scandlines Danmark ApS and Scandlines Deutschland GmbH v. Commission*, point 25.

34 Order of 10.09.2019, *Dansk Erhverv v. Commission*, point 19 (we underline).

35 Order of 6.11.2012, *Castelnou Energia v. Commission*, point 21.

36 Order of 30.11.2016, *Scandlines Danmark ApS and Scandlines Deutschland GmbH v. Commission*, points 33-37 (admitting German environmental NGO NABU’s intervention); Order of 10.09.2019, T-47/19, *Dansk Erhverv v. Commission*, ECLI:EU:T:2019:663, points 19-20 (admitting Danish environmental NGO Danmarks Naturfredningsforening’s intervention).

37 Order of 22.10.2019, C-175/19 P, *Stena Line Scandinavia AB v. Commission*, ECLI:EU:C:2019:1095, point 10.

38 Order of 22.10.2019, *Scandlines Danmark ApS and Scandlines Deutschland GmbH v. Commission*, C-174/19 P, ECLI:EU:C:2019:1096, point 57 (we underline).

39 Order of 30.11.2016, *Scandlines Danmark ApS and Scandlines Deutschland GmbH v. Commission*, point 16.

40 Jugement of 19.12.2013, *Vent de Colère e.a. v.* Commission, C-262/12, ECLI:EU:C:2013:851.

41 Milieu Consulting, “Study on EU implementation of the Aarhus Convention in the area of access to justice in environmental matters: Final report” (September 2019, 07.0203/2018/786407/SER/ENV.E.4), available at https://ec.europa.eu/environment/aarhus/pdf/Final_study_EU_implemention_environmental_matters_2019.pdf, pp. 106-107.

42 Ibid, pp. 170-171 and 175.

43 Ibid, pp. 131 and 171.

44 Ibid, pp. 170-171 and 175.

45 Commission Study on the enforcement rules and decisions of State aid by national courts (2019), ibid.

46 Council Regulation (EU) 2015/1589 of 13 July 2015 laying down detailed rules for the application of Art. 108 of the Treaty on the Functioning of the European Union.

47 Communication ACCC/C/2015/128, “EU statement following the Communicant’s 6.11.2020 Update concerning the judgment of the Court of Justice of the EU in Case C-594/18 P, Commission v Austria”, 7.12.2020, fn. 10.

48 See Study requested by the European Parliament JURI Committee, “Can Nature Get it Right” (PE 689.328-March 2021), authored by Jan Darpö. See also Krämer, L: Rights of nature and their implementation. Journal for European Environmental Planning Law (JEEPL) 2020, pp. 47-75.

49 European Commission report, “The costs of not implementing the environmental acquis” (ENV.G.1/FRA/2006/0073, September 2011), available online: https://ec.europa.eu/environment/enveco/economics_policy/pdf/report_sept2011.pdf.

50 Regulation (EC) No 1367/2006 of the European Parliament and of the Council of 6 September 2006 on the application of the provisions of the Aarhus Convention on Access to Information, Public Participation in Decision-making and Access to Justice in Environmental Matters to Community institutions and bodies, OJ L 264 of 25.9.2006, p. 13–19.

51 Under the Lisbon Treaty numbering, these provisions are now Art. 101, 102, 106 and 107 TFEU.

52 Commission decision of 08.10.2014 on the aid measure SA.34947 (2013/C) (ex 2013/N) which the United Kingdom is planning to implement for Support to the Hinkley Point C Nuclear Power Station, C(2014) 7142 final cor.

53 ACCC findings, paras 107-108.

54 Ibid, para. 106.

55 Ibid, paras 109-112.

56 Ibid, paras 95-96.

57 Ibid, para. 116 onwards.

58 Council Regulation (EU) 2015/1589 of 13 July 2015 laying down detailed rules for the application of Art. 108 of the Treaty on the Functioning of the European Union, Art. 4(4).

59 Ibid, Art. 6 and 7.

60 Since this is a matter of compliance of a Member State with EU law this does not fall under protection of business information. For an example of a State aid decision that merely mentions that the Member State should ensure compliance of the project with the EU Noise Directive, whereas that State was under an infringement procedure for mis-transposition of the directive, see Commission decision on State Aid SA.48472 (2018/N) – Croatia – Amended concession agreement relating to the Istrian Y Motorway. In the authors’ opinion, this circumstance should instead lead the Commission to presume that the activities on that Member State’s territory breach EU environmental law.

